# Multi-organ failure induced by Nivolumab in the context of allo-stem cell transplantation

**DOI:** 10.1186/s40164-019-0132-2

**Published:** 2019-03-28

**Authors:** Julie Charles, Diane Giovannini, Nicolas Terzi, Carole Schwebel, Nathalie Sturm, Dominique Masson, Marie-Thérèse Leccia, Jean-Yves Cahn, Olivier Manches, Claude-Eric Bulabois, Laurence Chaperot

**Affiliations:** 1 Institute for Advanced Biosciences, Université Grenoble Alpes, INSERM 1209, UMR CNRS 5309, Grenoble, France; 2 Dermatology Department, Grenoble Alpes University Hospital, Grenoble, France; 3 Medical Intensive Care Unit, Grenoble Alpes University Hospital, Grenoble, France; 4 Anatomic Pathology Department, Grenoble Alpes University Hospital, Grenoble, France; 5 Hematology Department, Grenoble Alpes University Hospital, Grenoble, France; 6 INSERM U1042, Grenoble, France; 7 INSERM U1039, Grenoble, France; 8 EFS-Auvergne Rhône-Alpes, Grenoble, France

**Keywords:** Hodgkin lymphoma, Nivolumab, PD1, GVHD, Myositis, Allogeneic stem cell transplantation, Immune-related adverse events

## Abstract

**Background:**

Immune checkpoint inhibitors have radically changed the landscape of anti-tumor therapies in several malignancies. However the adverse events associated with immune checkpoint blockade in combination with other treatments remains to be thoroughly documented. Here we report the case of a 33-year-old male with classical Hodgkin lymphoma who was successfully treated for lymphoma but experienced serious and eventually fatal multisystem organ failure following nivolumab administration and allogeneic stem cell transplantation.

**Case presentation:**

The patient was diagnosed with stage IIIa nodular sclerosing Hodgkin lymphoma. Originally treated by chemotherapy and autologous stem cell transplantation, he subsequently received two allogeneic stem cell transplants from matched and haplo-identical siblings upon successive disease recurrences. Nivolumab treatment was administered prior to the second allograft, after which complete remission of lymphoma was achieved (year 10), as evidenced by clinical and radiographic examination. However within the next 3 months, the patient went on to develop a constellation of symptoms affecting multiple organs, including acute pneumonia with no evidence of bacterial infection, widespread cutaneous eruptions on trunk and lower limbs, mucosal ulcerations, myositis, diarrhea and colitis. Further complications included hepatic cytolysis, acute renal failure, pancreatitis, as well as complete heart block. Some of these injuries being suggestive of graft-versus-host disease, the patient was administered immunosuppressive therapy (mycophenolate, steroids and polyvalent immunoglobulins), but died shortly afterwards. Tissue biopsies revealed extensive lymphocytic infiltration (mostly CD3 + T cells) in skin, liver, and most peculiarly in muscles, including the myocardium. Massive lymphoid-histiocytic infiltration of muscle fibers was accompanied by acute necrotizing myositis and endomysial inflammation.

**Conclusions:**

Multi-organ failure represents a rare but potentially fatal outcome of immune checkpoint blockade in patients receiving allogeneic stem cell grafts. Nivolumab may induce atypical immune-mediated tissue inflammation and damage, such as the extensive muscular polymyositis described here in a patient with Hodgkin lymphoma. Nivolumab might also worsen GVHD symptoms in the context of allogeneic stem cell transplantation. Irrespective of the actual pathological mechanisms, clinicians should be alerted to these fatal drug-related toxicities.

**Electronic supplementary material:**

The online version of this article (10.1186/s40164-019-0132-2) contains supplementary material, which is available to authorized users.

## Introduction

Standard front line treatments such as chemotherapy with or without radiotherapy can cure patients with classical Hodgkin Lymphoma (cHL), however 15–20% of patients relapse or become refractory to these treatments. High dose chemotherapy followed by autologous stem cell transplant (SCT) is usually proposed for chemosensitive relapse, with an approximate 50% response rate. For patients who relapse after autologous stem cell transplant, various options are possible: chemotherapy, radiotherapy, allogeneic SCT, or immune checkpoint blockers, as recently reviewed by Alinari et al. [1].

Allogeneic SCT with reduced-intensity conditioning regimen (RIC) is a validated approach to treat patients with chemoresistant relapse, relapse post autologous transplant, with cells from matched or mismatched unrelated donor, or haploidentical related donor. With these non-myeloablative allogeneic SCT, the 3-year overall survival is 63–76%, and the progression-free survival is 58–59% [2, 3]; the graft-versus-host disease (GVHD)-free relapse-free survival being longer with haplo-identical donor compared to mismatched unrelated donor or cord blood [3]. The rationale for allografting involves the graft-versus-tumor effect, and long-term anti-tumor responses rely on an immunological effect mediated by donor immune cells. The immune-mediated graft-versus-tumor effect is often associated with development of GVHD, GVHD being a major cause of non-relapse morbidity and mortality after allo-SCT.

Besides allo-SCT, targeting of the PD-1/PD-L1 axis represents an alternative strategy to manipulate the immune system in cHL [4]. Anti PD-1 is known to restore anti-tumor immune responses, and the clinical efficacy of this immune checkpoint blocker has been demonstrated in several cancers. PD-1-based therapy is particularly interesting in cHL because the predominant cells present within the tumor are mostly T-cells, along with macrophages, eosinophils, plasma cells, B cells, and neutrophils; whereas malignant Reed-Sternberg cells constitute less than 5% of the tumor mass. PD-1/PD-L1 interaction could contribute to immune subversion in cHL, since an increased amount of PD-1 + tumor-infiltrating lymphocytes is a negative prognostic factor of overall survival [5], while up-regulation of PD-L1 is observed on malignant cells and tumor infiltrating macrophages [6]. High expression of PD-L1 in cHL is frequently caused by alterations of the PD-L1 locus (9p24.1 amplification) that are associated with shorter progression free survival [7]. Nivolumab recently received accelerated approval by the U.S. Food and Drug Administration (FDA) for patients with cHL that relapsed or progressed after autologous SCT and post-transplantation brentuximab vedotin (CD30 mAb conjugated with a microtubule disrupting agent) [8]. Immune checkpoint blockers can induce immune-related adverse events such as dermatitis, endocrinopathies, hepatitis, colitis or pneumonitis. These toxic effects are thought to be mediated by autoreactive T cells no longer kept in check by feedback mechanisms. They are generally manageable with high dose glucocorticoids, but can be fatal in some cases. Very recently, two cases of fulminant myocarditis accompanied by myositis have been described in patients treated with a combination of anti PD-1 and anti-CTLA-4 [9].

Anti-PD-1 has been successfully used to treat patients with relapsed cHL after autologous-SCT [10, 11], but few patients were treated with immune checkpoint inhibitors after an allo-SCT [4, 12–16]. Indeed, there could be a risk of GVHD induction or exacerbation since PD-1/PD-L1 blockade may potentially trigger expansion and activation of allogeneic T cells [17, 18]. Younes et al. reported a clinical trial with 5 cHL patients who received allo-SCT after Nivolumab discontinuation; 3 of them developed acute GVHD, but were still alive at the time of analysis [11]. Several retrospective analyses documented the efficacy of PD-1 blockade after allo-SCT in cHL, although it was marred by the frequent onset of acute GVHD [4, 19, 20]. Thus, among 39 cHL patients who received allo-CST 7–260 days after anti-PD-1 treatment, high response rates were observed but immune-related toxicity led to 4 treatment-related deaths [20]. None of these latter reports documented any cardiac or endocrine pathology. We report here a case of fatal myocarditis induced by sequential treatment with PD-1 inhibitor Nivolumab and allo-SCT in a Hodgkin lymphoma patient.

## Case report

A 33-year-old patient was diagnosed in 2006 with stage IIIa nodular sclerosing Hodgkin lymphoma, treated with 12 different therapeutic regimens due to multiple relapses, including successively ABVD, radiotherapy, cisplatin containing regimen and autologous stem cell transplantation after BEAM conditioning regimen (year 4) (Additional file 1: Figure S1). He relapsed again, and was treated successively by bendamustin, FMS tyrosine kinase inhibitor, ICE, holoxan; bendamustin, and a first allogeneic stem cell transplantation from one of his HLA-matched sister (year 9) (conditioning regimen: Fludarabine 30 mg/m^2^ day 1–5; busulfan 3.2 mg/kg day 3 and 4; antithymocyte globulin 2.5 mg/kg day 5 and 6. 98% donor-type chimerism was achieved after this first allo-SCT.

He unfortunately relapsed, and successively received vinblastine, navelbin, brentuximab, gemcitabine, and 8 injections of Nivolumab every 2 weeks (3 mg/kg; pre-approval access, French authorization), from March to June 2016 (year 10) followed by a second allogeneic SCT from another HLA-haplo-identical sibling donor (conditioning: TBI 2 Gy on day 1; Cyclophosphamide 14.5 mg/kg/day day 2 and 3; Fludarabine 30 mg/m^2^/day, day 2 to 6). Full chimerism was reached 1 month after the haplo-SCT.

**Fig. 1:**
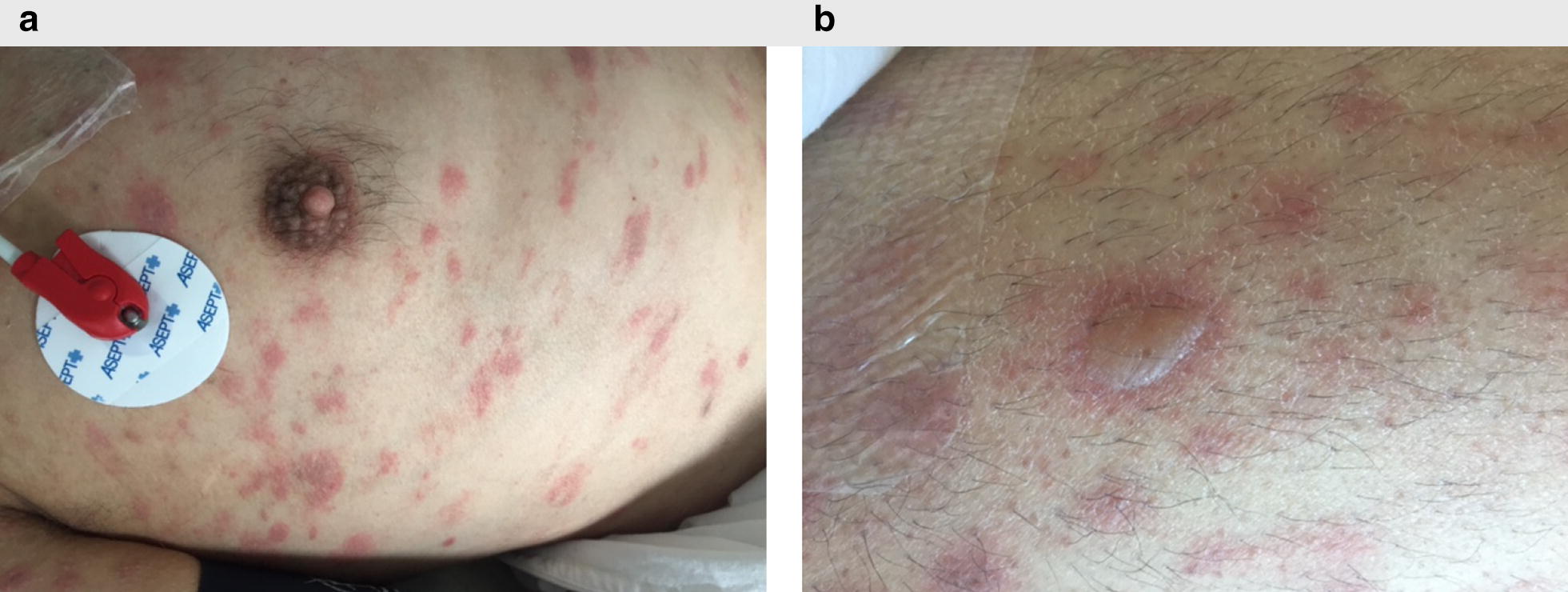
Skin lesions on trunk and upper limb. **a** Cutaneous eruption on trunk. **b** Vesiculo-bullous elements within scattered erythematous plaques, lacking Nikolsky’s sign, on trunk

Clinical and radiographic resolution of cHL were reached, but in October 2016, he was hospitalized for an acute interstitial pneumonia associated with hepatic cytolysis and cutaneous eruption consisting of interspersed erythematous plaques with central vesiculo-bullous elements lacking Nikolsky’s sign on trunk, shoulders, abdomen and legs. There were remaining patches of unaffected skin (Fig. 1). Mucosal (oral, ophthalmic and genital) lesions were also observed, with localized ulcerations and erosions. Bacteriological investigations including bronchoalveolar lavage were negative. Antibiotics were initiated but blood cultures returned negative. Subsequently, he presented with acute hypoventilation requiring invasive mechanical ventilation. Cerebral CT-scan and magnetic resonance imaging (MRI) were performed and did not reveal any cerebral abnormality. Lumbar puncture was performed, indicating normal white-cell count and protein/glucose levels. An electromyogram was performed, favoring myositis.

Hepatic cytolysis and cholestasis progressed (maximal values: ASAT: 942 UI/lL, × 25.4 normal range; ALAT: 1 166 UI/L, ×14.9 normal range; bilirubin 629 µmol/L, ×37 normal range). Further complications occurred, including acute renal failure requiring hemodialysis, diarrhea and moderate colitis. Although some of these adverse events were compatible with severe GVHD, others were uncommon and highly evocative of immune checkpoint blockers-induced immune-related adverse events.

**Fig. 2:**
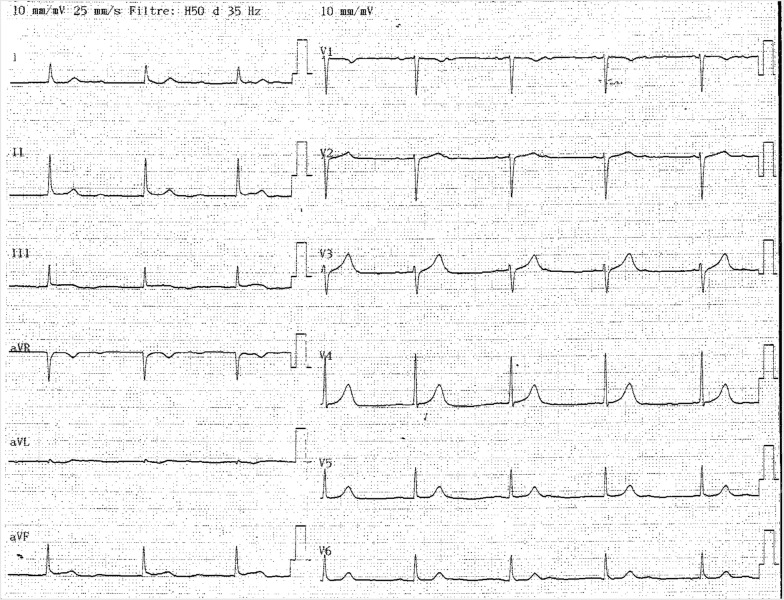
Progression to heart block. Electrocardiography showing regular rhythm at a rate of 55 bpm with normal QRS complex duration and morphology. AS atrial rate (70 bpm) was faster than the ventricular rate (55 bpm), and PP interval was constant with no relationship between the P waves and QRS, demonstrating atrioventricular dissociation

The patient exhibited endocrine complications, with acute pancreatitis (Balthazar C) and hypothyroidism (TSH 59 IU/L). Moreover, troponin was elevated (4 µg/L) without any evidence of myocardial infarction; ECG (Fig. 2) showed regular rhythm at a rate of 55 bpm with normal QRS complex duration (< 0.12 s) and morphology. There were regular atrial waveforms seen at a rate of 70 bpm, and of same morphology with an isoelectric baseline between each atrial waveform. PP interval was constant with no relationship between the P waves and QRS, demonstrating atrioventricular dissociation. AS atrial rate was faster (70 bpm) than the ventricular rate (55 bpm). Complete heart block was diagnosed while subsequent junctional escape rhythm axis was normal at +45° (positive and equivalent QRS voltage in leads I and aVF). Beat to beat QRS complex amplitude remained constant along the trace, T wave amplitude and morphology as well (no electric alternans). There was no argument for left ventricular hypertrophy as RV2 + SV5 < 35 mm. Slope of ST segment was normal (no down or up-sloping ST segment depression). The QT/QTc intervals were normal (600 ms/428 ms). Additional transthoracic echocardiography confirmed an acute myocarditis.

Eventually, these multisystemic injuries were hypothesized to result from cumulated immune-related adverse events induced by Nivolumab and/or exacerbated GVHD. The patient successively received mycophenolate mofetil, steroids (1 to 2 mg/kg) and polyvalent immunoglobulins, without any improvement leading to death.

**Fig. 3:**
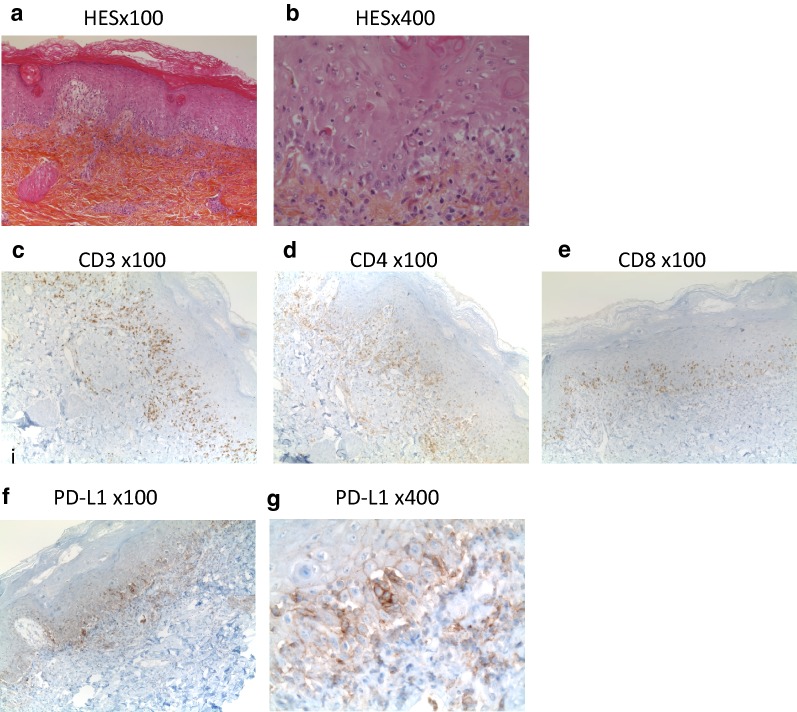
Extensive immune infiltrate in skin biopsies. Skin biopsies were fixed in formal acetic alcohol and included in paraffin. **a** HES staining at low magnification (×100). **b** HES at high magnification (×400), lymphocytes CD3 + (**c**), CD4 + (**d**), CD8 + (**e**) located along the dermoepidermal junction, at low magnification (×100) (**f**) and **g** immune cells and keratinocytes PD-L1 + at low (e ×100) and high magnification (f ×400)

A skin biopsy was analyzed by HES coloration (Fig. 3a, b) after hospitalization, 3 months after the second allograft. The stratum corneum was thickened, the epidermis contained diffused inflammatory cells, and there was an epithelial intercellular oedema. Numerous inflammatory cells accumulated along the dermoepidermal junction, leading to its liquefaction. Necrotic keratinocytes were frequently seen, without typical satellite cell necrosis. One section stained with periodic acid Schiff (PAS) did not show any infectious agent (not shown). Immunohistochemistry revealed the presence of numerous CD3 + lymphocytes, with similar proportions of CD4 + and CD8 + lymphocytes (Fig. 3c–e). PD-L1 was found to be expressed in the inflamed dermoepidermal junction and in the epidermis both on immune cells and on keratinocytes (Fig. 3f, g).

In the liver biopsy (collected 4 months after the second allograft) there was moderate inflammation, with discrete lymphocytic infiltration. An important cholestasis was seen, associated with foam cells. There was no vein endotheliitis. PD-L1 was found to be expressed on inflammatory cells, as well as on Kupffer and endothelial cells (Additional file 2: Figure S2).

**Fig. 4:**
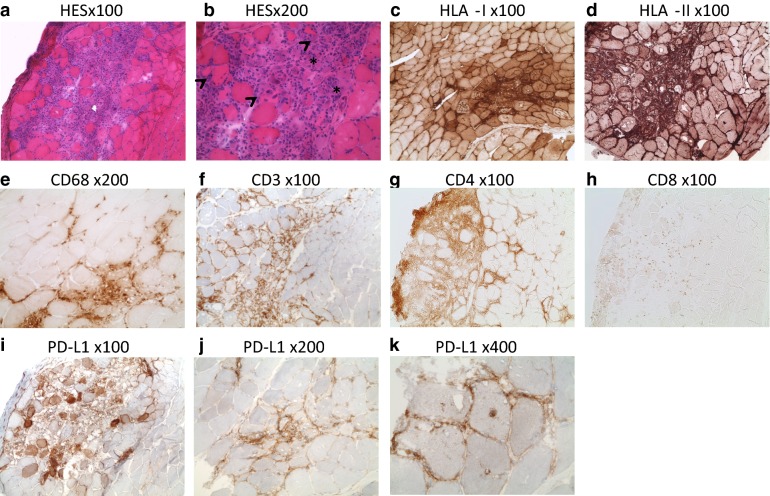
Immune infiltrate in muscle tissue. HES staining and immunohistochemistry analysis on muscle biopsy frozen in isopentan. **a** HES staining at low (×100) and **b** moderate magnification (×200) showing an important inflammatory endomysial infiltrate. Images of myophagia are seen. **c** HLA class I (×100) and **d** HLA class II (×100) stainings show a diffuse pattern with strong sarcoplasmic membrane positivity. Lymphocytic infiltration was also assessed. **e** Some CD68 + histiocytes are seen (×200), while a predominant CD3 + lymphocyte infiltration (×100) was observed (**f**), consisting mostly of CD4 + (×100) (**g**) but few CD8 + lymphocytes (**h** ×100). i) to k) PD-L1 expression is shown at low (**i** ×100), moderate (**j** ×200) and high magnification (**k** ×400). PD-L1 was found to be expressed in the inflamed area, both on immune cells and myocytes

A muscular biopsy was performed 3 months after the haplo-SCT, and showed foci of endomysial inflammation (Fig. 4a, b). There was evidence of acute necrotizing myositis characterized by several foci of necrotic muscle fibers, macrophage activity and some regenerative fibers (Fig. 4b arrows highlight some basophilic fibers with nuclear internalizations). There was massive lymphoid-histiocytic infiltration of muscle fibers (Fig. 4b). The infiltrating cells were mostly CD3 + , although some CD68 + macrophages were present (Fig. 4e, f). In the endomysium, CD4 + lymphocytes were in higher density than CD8 + (Fig. 4g, h). There was a high expression of both HLA class I and HLA class II molecules at the surface of infiltrating cells and a diffuse sarcolemic expression. The expression of both HLA molecules was also sarcoplasmic in the inflammatory foci (Fig. 4c, d), confirming the diagnosis of myositis. PD-L1 was found to be expressed in the inflamed area, both on immune cells and myocytes (Fig. 4i–k).

## Discussion

The patient we describe here was successively treated by Nivolumab and allogeneic haplo-identical stem cell transplantation, for classical Hodgkin disease. These treatments led to an efficient antitumor response, but also to severe and ultimately fatal adverse events that were evocative of a drug reaction driven by immune checkpoint blockade. The mean elimination half-life for Nivolumab being 26 days, it is likely that the drug was still present at the time of the graft, 1 month after the last injection. The patient displayed some classical manifestations of GVHD, characterized by diarrhea, hepatitis, and renal dysfunction. He also developed dermatitis, endocrine troubles, with pancreatitis and hypothyroidism, and muscular polymyositis due to muscle attack by lymphocytes, evoking immune checkpoint related toxicity. Multi-organ failure, associated with myocarditis and rhabdomyolysis, led to his death 5 months after allo-SCT.

Myositis is an uncommon manifestation of GVHD (12 cases of myositis in a series of 7161 patients who underwent HSCT [21]). Besides myositis, cardiomyolysis leading to heart failure is very rarely observed in the GVHD context [22–24]. Strikingly, myositis with fulminant myocarditis was recently described in two melanoma patients treated with immune checkpoint blockers [9]. In these latter cases, just as in the cases documented in the GVHD context and in the present report, there was massive infiltration of the myocardium by T lymphocytes and macrophages. The two melanoma patients mentioned above [9] and the patient we describe here shared the HLA-DQB1*03:01 allele, however this HLA- allele is very frequent, being expressed by more than 30% of Caucasian. In the muscle biopsy we analyzed, muscle fibers expressed high levels of HLA class I and class II molecules in the inflamed areas, suggesting that these cells could present antigens to infiltrating CD8 + and CD4 + T lymphocytes. It is striking that these attacks of muscle fibers or myocardium occur in the context of an ongoing antitumor response. One hypothesis is that some anti-tumor T cells clones could recognize an antigen also present in the skeletal muscle and/or the myocardium. Alternatively, the deleterious T cell clones may be specific for a tumor antigen that presents sufficient similarities to allow cross-recognition of a different muscle antigen. The onset of fatal cardiac toxicity has also been observed following administration of MAGE-A3 specific TCR-engineered T-cells [25]. The death of two patients occurred within 5 days after infusion of the anti-MAGE-A3 specific T cells, and it was shown that the myocardium was highly infiltrated by T cells cross-recognizing the cardiac Titin protein [26, 27]. All these case reports suggest that the development of cardiac toxicity must be acknowledged as a possible side effect during immunotherapy.

The mechanisms underlying this toxicity must be analyzed precisely, but the PD1/PD-L1 axis is likely to be involved. Although rare, fatal ICB-associated myocarditis is being increasingly reported in a wide spectrum of cancer types [28]. While PD-1 expression is low in resting T cells, it is inducible following T cell activation, and also expressed by activated B cells and monocytes. PD-L1 is upregulated upon inflammation, and its expression has been evidenced on both CD4 and CD8 T cells in GVHD patients [29], where its expression could modulate T cell mediated allo-responses. However, the number and nature of the affected organs, such as the heart, is very unusual even for patients who receive two or three allo-SCT (Duncan, Biol Blood Marrow Transplant 21 (2015) 151e158)(Spitzer, Biol Blood Marrow Transplant 22 (2016) 1449-1454). A link of causality between Nivolumab administration and the fatal side-effects is difficult to ascertain, however the symptoms experienced by the patient are extremely rare in the context of GVHD (such as thyroidism, myocarditis or pancreatitis), whereas they have been described in an increasing number of cases upon Nivolumab administration as a single agent [28, 30]. We thus favor the hypothesis that the multiorgan attack was triggered by Nivolumab, owing to the similarities with the described immune-related adverse events of Nivolumab and the dissimilarities with classical GVHD symptoms. It is also possible that blocking PD-1 helped worsen and extend the alloreactivity after the second transplant [5, 11, 20]. Interestingly, in murine GVHD models the heart was protected from alloreactive T cells by up-regulation of PD-L1 on immune and endothelial cells [31], whereas PD-L1 knock-out led to myocarditis triggered by autoimmune T cells [32]. We observed a high expression of PD-L1 in the skin and inflamed muscle biopsy, probably induced by inflammatory cytokines secreted in the micro-environment. However, despite the presence of this co-inhibitory molecule, the damaging immune activation was not blocked, suggesting that Nivolumab administration may have hampered its inhibitory and tissue-protective function. Interestingly, immune profiling of lymphoma patients treated with Nivolumab prior to allo-SCT has shown a long-lasting depletion of PD1 + T lymphocytes, persisting at least 6 months after SCT [20]. Immune-checkpoint blockers-induced multi-organ failure remains a rare event affecting patients receiving allogeneic stem cell transplantation. These fatal immune-related adverse events can occur several weeks after treatment discontinuation. Irrespective of the actual pathological mechanisms, clinicians should be vigilant and diagnose as early as possible this potentially fatal drug-related toxicity.

## Additional files

**Additional file 1: Figure S1.** Simplified timeline of clinical events and treatments.

**Additional file 2: Figure S2.** PD-L1 staining on liver biopsies is shown at low (left—×100), moderate (middle—200×) and high magnification (right—400×), with scattered expression in the cytoplasm of some hepatocytes and sinusoïdal expression.

## Abbreviations

cHL: classical Hodgkin lymphoma
GVHD: graft-versus-host disease
SCT: Stem cell transplant
RIC: reduced-intensity conditioning regimen

## References

[1] Alinari L, Blum KA (2016). How I treat relapsed classical Hodgkin lymphoma after autologous stem cell transplant. Blood.

[2] Castagna L, Bramanti S, Devillier R, Sarina B, Crocchiolo R, Furst S (2017). Haploidentical transplantation with post-infusion cyclophosphamide in advanced Hodgkin lymphoma. Bone Marrow Transplant..

[3] Gauthier J, Castagna L, Garnier F, Guillaume T, Socie G, Maury S (2017). Reduced-intensity and non-myeloablative allogeneic stem cell transplantation from alternative HLA-mismatched donors for Hodgkin lymphoma: a study by the French Society of Bone Marrow Transplantation and Cellular Therapy. Bone Marrow Transplant.

[4] Herbaux C, Gauthier J, Brice P, Drumez E, Ysebaert L, Doyen H (2017). Efficacy and tolerability of nivolumab after allogeneic transplantation for relapsed Hodgkin lymphoma. Blood.

[5] Muenst S, Hoeller S, Dirnhofer S, Tzankov A (2009). Increased programmed death-1 + tumor-infiltrating lymphocytes in classical Hodgkin lymphoma substantiate reduced overall survival. Hum Pathol.

[6] Chen BJ, Chapuy B, Ouyang J, Sun HH, Roemer MG, Xu ML (2013). PD-L1 expression is characteristic of a subset of aggressive B-cell lymphomas and virus-associated malignancies. Clin Cancer Res.

[7] Roemer MG, Advani RH, Ligon AH, Natkunam Y, Redd RA, Homer H (2016). PD-L1 and PD-L2 Genetic Alterations Define Classical Hodgkin Lymphoma and Predict Outcome. J Clin Oncol.

[8] Ansell SM (2017). Nivolumab in the treatment of Hodgkin Lymphoma. Clin Cancer Res..

[9] Johnson DB, Balko JM, Compton ML, Chalkias S, Gorham J, Xu Y (2016). Fulminant myocarditis with combination immune checkpoint blockade. N Engl J Med.

[10] Ansell SM, Lesokhin AM, Borrello I, Halwani A, Scott EC, Gutierrez M (2015). PD-1 blockade with nivolumab in relapsed or refractory Hodgkin’s lymphoma. N Engl J Med.

[11] Younes A, Santoro A, Shipp M, Zinzani PL, Timmerman JM, Ansell S (2016). Nivolumab for classical Hodgkin’s lymphoma after failure of both autologous stem-cell transplantation and brentuximab vedotin: a multicentre, multicohort, single-arm phase 2 trial. Lancet Oncol.

[12] Villasboas JC, Ansell SM, Witzig TE (2016). Targeting the PD-1 pathway in patients with relapsed classic Hodgkin lymphoma following allogeneic stem cell transplant is safe and effective. Oncotarget..

[13] Yared JA, Hardy N, Singh Z, Hajj S, Badros AZ, Kocoglu M (2016). Major clinical response to nivolumab in relapsed/refractory Hodgkin lymphoma after allogeneic stem cell transplantation. Bone Marrow Transplant.

[14] Angenendt L, Schliemann C, Lutz M, Rebber E, Schulze AB, Weckesser M (2016). Nivolumab in a patient with refractory Hodgkin’s lymphoma after allogeneic stem cell transplantation. Bone Marrow Transplant.

[15] Onizuka M, Kojima M, Matsui K, Machida S, Toyosaki M, Aoyama Y (2017). Successful treatment with low-dose nivolumab in refractory Hodgkin lymphoma after allogeneic stem cell transplantation. Int J Hematol..

[16] Singh AK, Porrata LF, Aljitawi O, Lin T, Shune L, Ganguly S (2016). Fatal GvHD induced by PD-1 inhibitor pembrolizumab in a patient with Hodgkin’s lymphoma. Bone Marrow Transplant.

[17] Oshima Y, Tanimoto T, Yuji K, Tojo A (2017). Association between GvHD and nivolumab in the FDA adverse event reporting system. Bone Marrow Transplant.

[18] Blazar BR, Carreno BM, Panoskaltsis-Mortari A, Carter L, Iwai Y, Yagita H (2003). Blockade of programmed death-1 engagement accelerates graft-versus-host disease lethality by an IFN-gamma-dependent mechanism. J Immunol..

[19] Haverkos BM, Schowinksy J, Kaplan J, Kamdar M, Kanate AS, Saad A, et al. Checkpoint blockade for treatment of relapsed lymphoma following allogeneic hematopoietic cell transplant: use may be complicated by onset of severe acute graft versus host disease. Blood. 2016; 128: 1163. http://www.bloodjournal.org/content/128/22/1163.

[20] Merryman RW, Kim HT, Zinzani PL, Carlo-Stella C, Ansell SM, Perales MA (2017). Safety and efficacy of allogeneic hematopoietic stem cell transplant after PD-1 blockade in relapsed/refractory lymphoma. Blood.

[21] Stevens AM, Sullivan KM, Nelson JL (2003). Polymyositis as a manifestation of chronic graft-versus-host disease. Rheumatology (Oxford).

[22] Platzbecker U, Klingel K, Thiede C, Freiberg-Richter J, Schuh D, Ehninger G (2001). Acute heart failure after allogeneic blood stem cell transplantation due to massive myocardial infiltration by cytotoxic T cells of donor origin. Bone Marrow Transplant.

[23] Rackley C, Schultz KR, Goldman FD, Chan KW, Serrano A, Hulse JE (2005). Cardiac manifestations of graft-versus-host disease. Biol Blood Marrow Transplant.

[24] Rouah E, Gruber R, Shearer W, Armstrong D, Hawkins EP (1988). Graft-versus-host disease in the central nervous system. A real entity?. Am J Clin Pathol..

[25] Linette GP, Stadtmauer EA, Maus MV, Rapoport AP, Levine BL, Emery L (2013). Cardiovascular toxicity and titin cross-reactivity of affinity-enhanced T cells in myeloma and melanoma. Blood.

[26] Raman MC, Rizkallah PJ, Simmons R, Donnellan Z, Dukes J, Bossi G (2016). Direct molecular mimicry enables off-target cardiovascular toxicity by an enhanced affinity TCR designed for cancer immunotherapy. Sci Rep..

[27] Cameron BJ, Gerry AB, Dukes J, Harper JV, Kannan V, Bianchi FC (2013). Identification of a Titin-derived HLA-A1-presented peptide as a cross-reactive target for engineered MAGE A3-directed T cells. Sci Transl Med..

[28] Moslehi JJ, Salem JE, Sosman JA, Lebrun-Vignes B, Johnson DB (2018). Increased reporting of fatal immune checkpoint inhibitor-associated myocarditis. Lancet..

[29] Saha A, O’Connor RS, Thangavelu G, Lovitch SB, Dandamudi DB, Wilson CB (2016). Programmed death ligand-1 expression on donor T cells drives graft-versus-host disease lethality. J Clin Invest..

[30] Boutros C, Tarhini A, Routier E, Lambotte O, Ladurie FL, Carbonnel F (2016). Safety profiles of anti-CTLA-4 and anti-PD-1 antibodies alone and in combination. Nature reviews Clinical oncology..

[31] Schilbach K, Schick J, Wehrmann M, Wollny G, Simon P, Schlegel PG (2007). PD-1-PD-L1 pathway is involved in suppressing alloreactivity of heart infiltrating t cells during murine gvhd across minor histocompatibility antigen barriers. Transplantation.

[32] Juchem KW, Sacirbegovic F, Zhang C, Sharpe AH, Russell K, McNiff JM (2018). PD-L1 prevents the development of autoimmune heart disease in graft-versus-host disease. J Immunol..

